# Risk and prognosis of secondary malignant neoplasms after radiation therapy for bladder cancer: A large population-based cohort study

**DOI:** 10.3389/fonc.2022.953615

**Published:** 2022-11-16

**Authors:** Ru Chen, Xiangpeng Zhan, Haoxin Jiang, Yang Liu, Zhi Jiang, Ming Jiang, Wen Deng, Xiaoqiang Liu, Guoxian Chen, Bin Fu

**Affiliations:** ^1^ Department of Urology, The First Hospital of Putian City, Putian, Fujian, China; ^2^ Department of Urology, The First Affiliated Hospital of Nanchang University, Nanchang, Jiangxi, China; ^3^ Department of Cardiology, The Second Affiliated Hospital of Nanchang University, Nanchang, Jiangxi, China; ^4^ Department of Thoracic Surgery, The First Affiliated Hospital of Nanchang University, Nangchang, China

**Keywords:** bladder cancer, radiation, secondary malignant neoplasms, risk, prognosis

## Abstract

**Objective:**

To investigate the association between radiotherapy and the risk of second malignant neoplasm (SMN) development among patients with bladder cancer (BC). Overall survival (OS) is compared among patients developing SMN and without.

**Method:**

We identified patients diagnosed with BC from the Surveillance, Epidemiology, and End Results (SEER) database. The development of an SMN is defined as any SMN occurring more than 5 years after the diagnosis of BC. The Fine-Gray competing risk regression is used to estimate the probability of SMN. The radiotherapy-associated risk (RR) for SMNs is assessed by Poisson regression. The Kaplan–Meier method was used to evaluate the OS of patients with SMNs. Propensity score matching (PSM) is performed.

**Results:**

A total of 76575 BC patients are enrolled in our study. The probability of SMNs in the radiotherapy cohort is statistically higher than in the non-radiotherapy cohort. In competing risk regression analysis, radiotherapy is proven to be associated with a higher risk of SMN (Hazard ratio: 1.23; 95% CI: 1.102–1.368). The radiotherapy-associated risks significantly increase in the radiotherapy cohort (RR: 1.28; 95% CI: 1.14–1.43). In site-specific analysis, statistically significant results are observed in lung and bronchus (LAB) cancer and hematological malignancies. The OS rate in patients developing SMN is significantly lower than that among matched patients with primary BC.

**Conclusion:**

Radiotherapy for BC is associated with SMN. Radiotherapy increases the risk of secondary low-dose area cancer development, including LAB cancer or hematological malignancies. Notably, this effect is not observed in the high-dose area involving pelvic tumors. Patients developing SMN showed poorer OS.

## Introduction

Urothelial bladder cancer (UBC) is the most common cancer of the urinary system (1). About one-third of patients are diagnosed with muscle-invasive bladder cancer (MIBC). In recent decades, radiotherapy is still an important adjuvant therapy for MIBC patients. First, radiotherapy is the central part of trimodal therapy, which has long been proposed by multiple teams and recommended as an alternative for carefully selected patients with MIBC (T2-T3 N0M0) (2). In addition, RT can be regarded as the adjuvant therapy after radical cystectomy (RC) in pathologically high-risk patients with local recurrence (3).

Despite the progress of RT, pelvic toxicity is still significant. The risk of developing secondary primary cancer (SPC) is a rare but noteworthy form of advanced toxicity. A body of previous studies has proposed that radiotherapy for pelvis organs will increase the risk of SPC (4, 5). For instance, data from the SEER database suggested that radiotherapy for rectal cancer increased the risk of developing uterine and ovarian cancer (5). A similar study investigated the association between radiotherapy and SPC in the pelvis and found that patients receiving pelvic radiotherapy have an increased risk of developing SPC (6). However, the results are inconsistent regarding the effect of RT from several studies. Warschkow et al. found that the overall risk of the SPC after pelvic radiotherapy was reduced, which was attributed to the decrease in prostate cancer after pelvic radiotherapy (4).

UBC as a pelvic organ is usually treated as a second malignant tumor caused by radiotherapy for other pelvic organs (6, 7). There is still a lack of studies focusing on the association between radiotherapy and SPC for bladder cancer. It is mainly because radiotherapy is more considered as an adjuvant treatment for MIBC or as an alternative treatment for patients who are not suitable for RC (1). In recent years, with the development of management for BC patients, the survival of BC, especially MIBC, has been significantly improved (8). A reported data showed that the 5-year overall survival rate of MIBC patients is approximately 60% to 70% after active treatment (8). Longer survival allows us to consider the potential effect caused by radiotherapy on the survival outcome. Therefore, we used the National Cancer Institute’s Surveillance, Epidemiology and End Results (SEER) database to investigate the effect of radiotherapy on the risk of subsequent SPC in BC patients and to assess their survival outcome.

## Methods

### Database and study population

**Figure 1:**
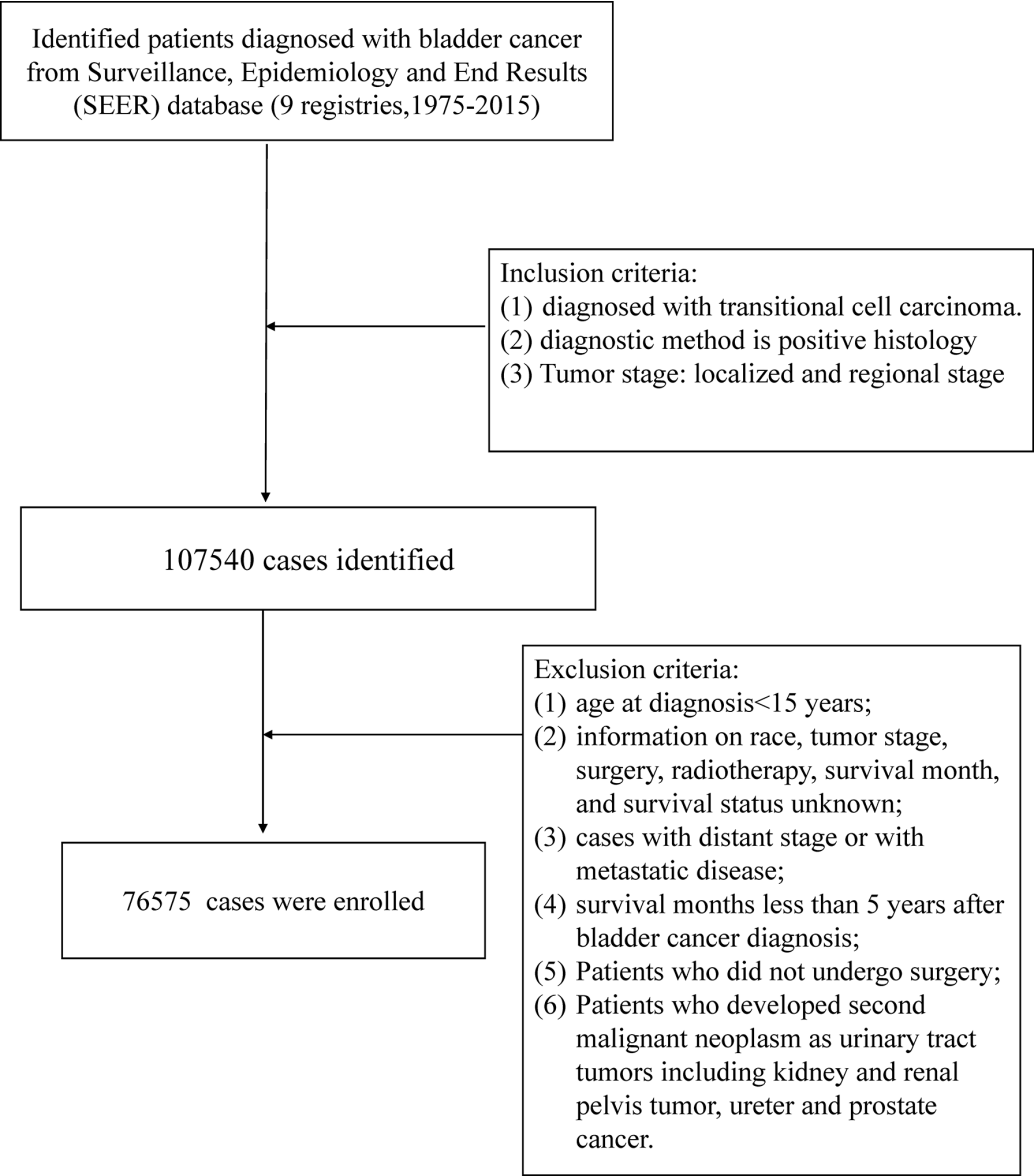
Flow chart for screening patients with bladder cancer treated with radiotherapy.

We identified patients diagnosed with bladder cancer as the first primary malignant tumor from the SEER database (nine registries) between January 1, 1975, and December 31, 2015 (ID: 20420-Nov2020). We searched all cancer sites based on the case list of “*The International Classification of Diseases for Oncology, Third Edition (Site recode ICD-O-3)*”. The study population was limited to cases pathologically diagnosed with transitional cell carcinoma (TCC), and the method of diagnostic confirmation was positive histology. We restricted the tumor stage to the localized and regional stage. Localized referred to a tumor confined entirely to the mucosa of the bladder, and a tumor staged as regional was defined as extending to surrounding organs, tissues, or regional lymph nodes. The exclusion criteria were as follows: (1) age at diagnosis <15 years; (2) information on race, tumor stage, surgery, radiotherapy, survival month, and survival status unknown; (3) cases with distant stage or with metastatic disease; (4) survival months less than 5 years after bladder cancer diagnosis; (5) patients who did not undergo surgery; (6) patients who developed a second malignant neoplasm as urinary tract tumors including kidney and renal pelvis tumors, and ureter and prostate cancer. Considering the connectivity of the urinary tract, the tumor might metastasize along the urinary tract, and the development of the secondary malignant neoplasm might not be the effect of radiotherapy (**Figure 1**).

### Treatment interventions for bladder cancer

We divided the study population into two groups according to the initial treatment modality. The radiotherapy group was defined as patients who received surgery and external-beam radiotherapy. The non-radiotherapy group comprised patients who underwent surgery but without radiotherapy. Notably, patients who received the radiotherapy coded with combination radiotherapy, radiation with an unknown method or source, radioactive implants, and radioisotopes were excluded from our study.

### Definition of outcome

The primary outcome of this study was the development of a second malignancy, and the positive outcome was defined as developing any type of malignant tumor, except a urinary tract tumor, with more than 5 years of follow-up after the diagnosis of BC considering the incubation period of at least 5 years from radiation exposure to the solid tumor (9). A history of cancer was obtained according to the case list of “*Sequence number*” which presented the order of all reportable primary tumors in the patient’s life. The SEER program followed the guidelines of the third edition of the *International Classification of Oncology Diseases* to distinguish between SPMN and recurrent diseases. In addition, we checked the histological behavior of SMN and excluded patients with transitional cell carcinoma. We first comprehensively evaluated the risk of developing second malignant neoplasms (SMN) by regarding all types of developed SMNs as the outcome. Then, we classified the developed secondary malignant tumors into five categories according to the location of occurrence in the human body: intrathoracic tumor [lung and bronchus (LAB) cancer and other intrathoracic tumors], hematological malignancies (leukemia and lymphoma), abdominal tumors (stomach, pancreas, liver, small intestine, colon except for rectum), pelvic tumors (rectum, uterus, and ovary cancer), and other tumors. The SMN follow-up began 5 years after the diagnosis of bladder cancer and ended on the date of diagnosis of any SMN, all-cause death, or the last follow-up.

### Statistical analysis

The composition ratio between RT and non-RT groups described the baseline characteristic distribution. Two-sample *t*-tests and chi-square test were utilized for continuous variable and categorical variables, respectively. The Fine-Gray competing risk regression model was utilized to evaluate the risk of SMN development and plot the probability of SMN curves. Meanwhile, the model was performed to adjust for the confounding factors, including age, sex, race, tumor stage, and chemotherapy received. The occurrence of a non-SMN (SMN defined as outcome event) or death from all-cause was identified as competing events. The results were presented as hazard ratios (HRs) and 95% confidence intervals (CI). The Poisson regression analysis was used to estimate the radiotherapy-associated risk (RR) and 95% CIs of SMN development between the radiotherapy cohort and non-radiotherapy cohort. Survival analyses were performed with the Kaplan–Meier method and log-rank tests to assess overall survival (OS) between patients with SMN and patients with primary BC. Only primary BC was defined as the only cancer that patients suffered from in their life. Propensity score matching was applied to adjust the potential baseline that matched 1:1 for survival comparison (caliper set at 0.02).

All statistical analyses were performed using the R software (version4.1.3). p-Values less than 0.05 were considered statistically significant.

## Results

### Patient characteristics

Finally, 76,575 BC patients were included in our study (**Table 1**). There were 2,145 cases and 74,430 cases in the radiotherapy cohort and non- radiotherapy cohort, respectively. Compared with those without radiotherapy, the radiotherapy cohort showed a higher proportion of patients aged over 50 years (p < 0.001). Patients in the radiotherapy cohort had a higher rate of Grade III/IV (77.3% vs. 29.4%; p < 0.001) and regional stage (65.4% vs. 9.1%; p < 0.001) compared to cases in the non- radiotherapy cohort. Chemotherapy was more common in the radiotherapy cohort (30.3% vs. 10.5%; *P*<0.001). Patients who received radiotherapy had a relatively shorter survival time than those who did not (mean survival months: 153 vs. 160, median: 127 vs. 137; p < 0.001). A total of 610 (27.85%) and 20,268 (25.72%) patients developed SMN in the radiotherapy cohort and non- radiotherapy cohort, respectively, through the follow-up period (**Table 1**).

**Table 1:** Comparisons of baseline characteristics of patients with bladder cancer by treatment modality.

	No. (%)		
Characteristic	Radiotherapy (2145)	No radiotherapy (74430)	p-Value
**Age at diagnosis**			<0.001^※^
<50 years	107 (5.0%)	7194 (9.7%)	
50–70 years	1142 (53.2%)	36962 (49.7%)	
>70 years	896 (41.8%)	30274 (40.7%)	
**Sex**			0.856
Female	535 (24.9%)	18710 (25.1%)	
Male	1610 (75.1%)	55720 (74.9%)	
**Race**			<0.001^※^
White	1935 (90.2%)	69069 (92.8%)	
Black	100 (4.7%)	2465 (3.3%)	
Other^a^	110 (5.1%)	2896 (3.9%)	
**Year of diagnosis**			
Median [IQR]	1980 [1980, 2010]	2000 [1980, 2010]	<0.001^※^
1975–1984	1123 (52.4%)	13675 (18.4%)	
1985–1994	415 (19.3%)	18974 (25.5%)	
1995–2004	329 (15.3%)	22035 (29.6%)	
≥2005	278 (13.0%)	19746 (26.5%)	
**Tumor grade**			<0.001^※^
Grade I/II	364 (17.0%)	44906 (60.3%)	
Grade III/IV	1659 (77.3%)	21880 (29.4%)	
Unknown	122 (5.7%)	7644 (10.3%)	
**Tumor stage**			<0.001^※^
Localized	743 (34.6%)	67644 (90.9%)	
Regional	1402 (65.4%)	6786 (9.1%)	
**Chemotherapy**			<0.001^※^
No	1494 (69.7%)	66618 (89.5%)	
Yes	651 (30.3%)	7812 (10.5%)	
**Survival month**			<0.001^※^
Mean	153	160	
Median [IQR]	127 [60.0, 471]	137 [60.0, 516]	
**Patients who developed SMN**	610(27.85%)	20268 (25.72%)	<0.001^※^

a Other includes American Indian/AK Native, Asian/Pacific Islander.

IQR, interquartile range; SMN, second malignant neoplasm.

^※^Statistically significant.

### Probability of SMNs

**Figure 2:**
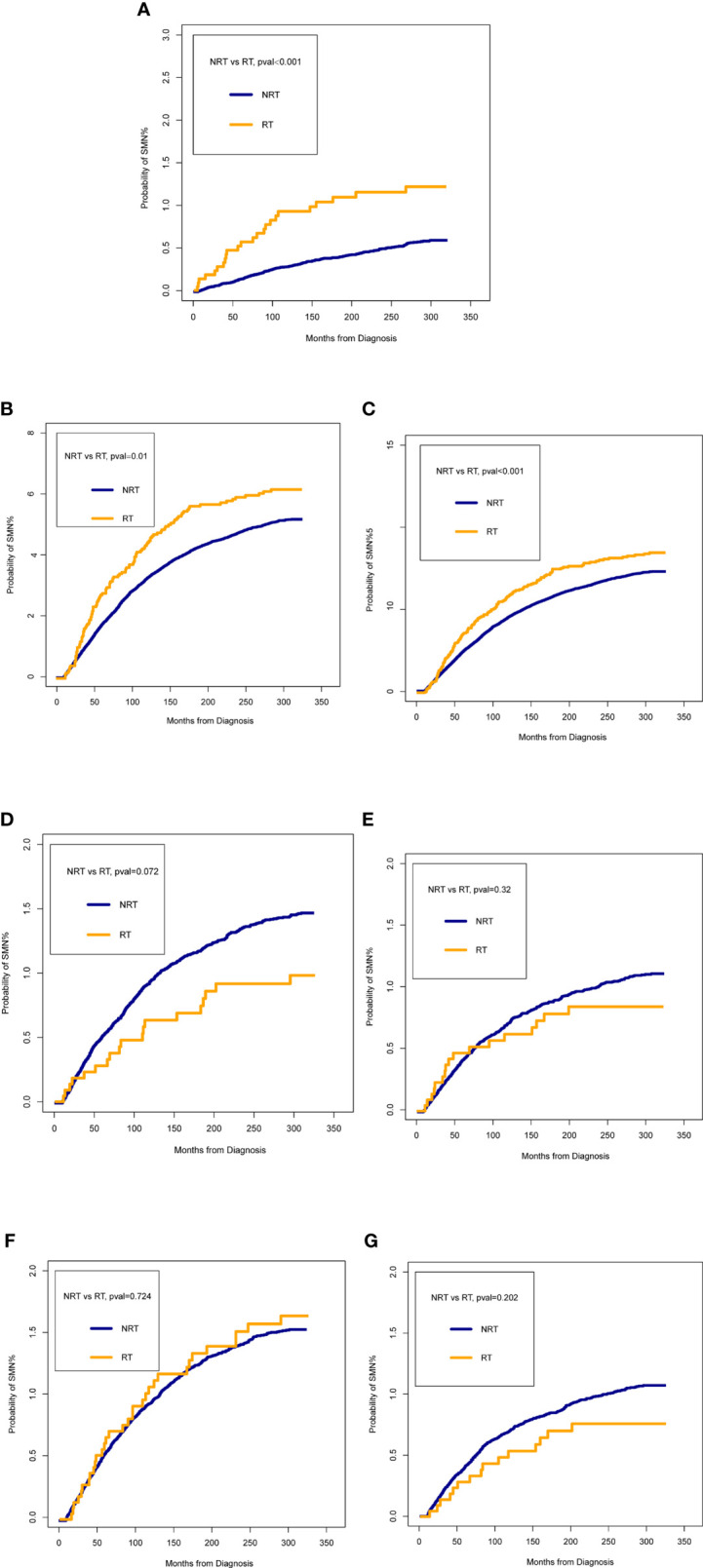
Comparisons of the probability of second malignant neoplasm (SMN) between patients who received radiotherapy (RT) and patients who did not receive RT. **(A)** all SMNs; **(B)** lung and bronchus cancer; **(C)** hematological malignancies; **(D)** other intrathoracic tumors; **(E)** abdominal tumors; **(F)** pelvic tumors; **(G)** other cancers. The time on the x-axis begins 60 months after bladder cancer diagnosis.

**Figure 2** presented the probability of combined SMNs by whether they were receiving radiotherapy after BC diagnosis. Of all SMNs, the probability in patients receiving radiotherapy was significantly higher than those without (p < 0.001) (**Figure 2A**). In the analyses of each type of SMN, the probability of LAB cancer and hematological malignancies were statistically higher in the radiotherapy cohort when compared with the non-radiotherapy cohort (all p < 0.05) (**Figures 2B, C**). No statistical differences were observed for other intrathoracic tumors, abdominal tumors, pelvic tumors, and other tumors analyzed between patients with and without radiotherapy (**Figures 2D–G**).

### Risk of SMNs associated with radiotherapy

After adjusting for the confounding factors, multivariable competing risk regression analysis suggested that receiving radiotherapy was associated with a higher risk of developing SMN (HR: 1.23; 95% CI: 1.102–1.368; p = 0.002) (**Table 2**). In SMN-specific analyses, a higher risk was obtained in LAB cancer (HR: 1.49; 95% CI: 1.27–1.76; p < 0.001) and hematological malignancies (HR: 1.51; 95% CI: 1.291–1.75; p < 0.001). We did not obtain statistically significant results for other intrathoracic, abdominal, pelvic, and other cancers.

**Table 2:** Risk of developing SMNs in patients with bladder cancer by statistical method.

	Multivariable competing riskregression (RT vs. NRT)	p-Value	Poisson regression(RT vs. NRT)	p-Value
SMN	Adjusted HR (95% CI)		Adjusted RR (95% CI)	
Developing SMN	1.23 (1.102–1.368)	P = 0.002^※^	1.28 (1.14–1.43)	P < 0.001^※^
Developing lung and bronchus cancer	1.49 (1.27–1.76)	P < 0.001^※^	1.41 (1.19– 1.67)	P < 0.001^※^
Developing hematological malignancies	1.51 (1.291–1.75)	P < 0.001^※^	1.67 (1.23–2.21)	P = 0.003^※^
Developing other intrathoracic tumors	0.737 (0.491–1.105)	P = 0.22	0.97 (0.48–1.73)	P = 0.93
Developing abdominal tumors	1.146 (0.754–1.74)	P = 0.59	1.07 (0.67–1.62)	P = 0.785
Developing pelvic tumors	1.339 (0.98–1.823)	P = 0.12	1.22 (0.85–1.7)	P = 0.34
Developing other cancers	0.885 (0.566–1.38)	P = 0.2	0.99 (0.61– 1.52)	P = 0.99

SMN, second malignant neoplasm; RT, radiotherapy; NRT, non- radiotherapy; HR, hazard ratio; RR, radiotherapy-associated risk.

^※^Statistically significant.

RRs were calculated to confirm the risk of SMNs associated with radiotherapy (**Table 2**). Similarly, multivariable Poisson regression analysis revealed that the additional risk for developing SMN attributable to radiotherapy was 1.28 (95% CI: 1.14–1.43; p < 0.001). A similarly increasing risk was observed for LAB cancer (RR: 1.41; 95% CI:1.19–1.67; p < 0.001) and hematological malignancies (RR: 1.67, 95% CI:1.23–2.21, p = 0.003).

### Survival outcome after SMN

**Figure 3:**
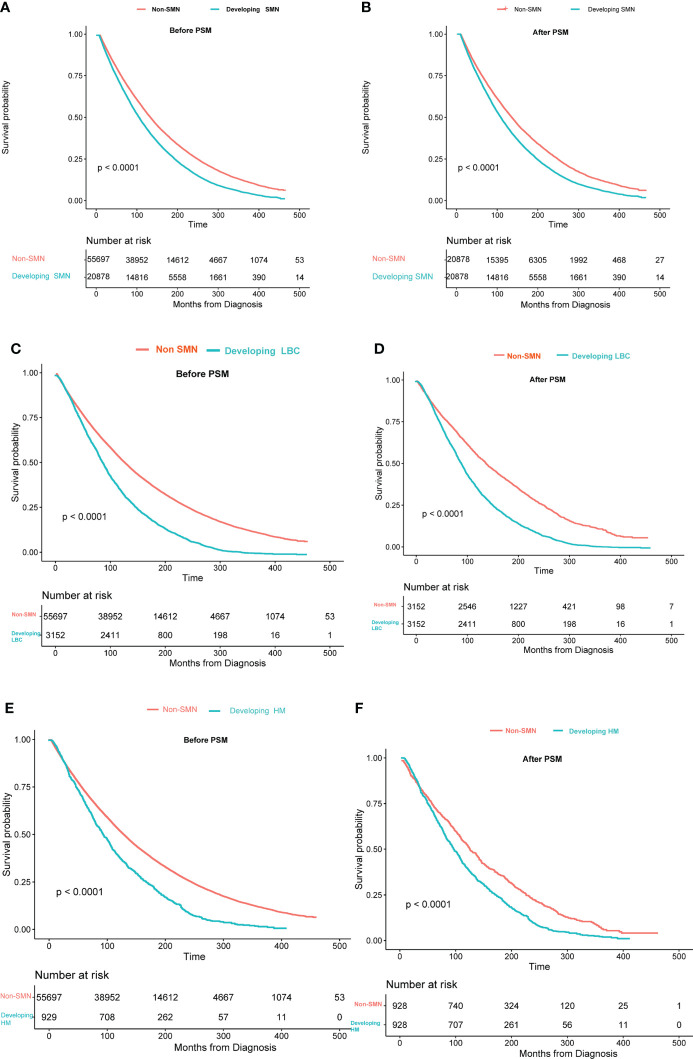
Overall survival between patients with only bladder cancer and developing second malignant neoplasm: **(A)** all SMNs before PSM; **(B)** all SMNs after PSM; **(C)** lung and bronchus cancer before PSM; **(D)** lung and bronchus cancer after PSM; **(E)** hematological malignancies before PSM;**(F)** hematological malignancies after PSM. The time on the x-axis begins 60 months after bladder cancer diagnosis.

We compared the OS of patients with only primary tumor and patients with SMN. Survival curves revealed that patients with only primary tumors had a significantly better OS than those with SMN (**Figure 3A**). Compared with those developing LAB or hematological malignancies, patients with only primary tumor showed a better prognosis (**Figures 3C, E**). After 1:1 PSM adjusting for the confounding factors, we observed similar results as before PSM (**Figures 3B, D, F**).

## Discussion

To the best of our knowledge, this is the first large population-based study to comprehensively evaluate the risk of developing SMN among bladder cancer survivors and to assess the survival outcome of SMN. We found that patients receiving radiotherapy showed a higher risk of developing SMN than those without. In the site-specific analysis, we observed the increasing risk of LAB cancer and hematological malignancies. Notably, we did not obtain a statistical difference in the risk of developing pelvic tumors between patients receiving radiotherapy and without. Then, we observed a poorer OS rate in BC patients developing SMN, LAB cancer, or hematological malignancies compared with those with only primary cancer.

Radiotherapy includes accidental exposure to the surrounding normal tissue, thereby increasing the risk of radiation-induced second cancer for many cancers (10). A relatively convincing explanation for the SMN caused by radiotherapy was that low-dose radiation could cause base damage, single-strand DNA breaks, and double-strand breaks (DSBs) (11). DSB can cause gene mutation then the following transformation from the radiated cell to a malignant cell. In addition, the DNA repair proteins, which usually protect DNA from damage, might be damaged, resulting in the increasing risk of SMN.

Radiotherapy-related SMN can be found in the organs within the radiotherapy range (high dose area) or organs far beyond the radiation range (low dose area). In the long-term follow-up of A-bomb survivors in Hiroshima and Nagasaki, reported data showed the increased rate of leukemia and solid tumors in the population. Moreover, lung cancer is the most common solid tumor in the survivors (11). These results are one of the most conclusive evidence of radiation-induced SMN. Similarly, a study based on the SEER and UK population revealed that lung cancer accounts for the most significant SMN caused by radiotherapy (23.7% of the total) (10). In this study, we also found a significantly higher proportion of patients developing LAB cancer or hematological malignancies in the cohort of radiotherapy. However, the difference in the risk of developing SMN was not found in other low-dose areas. The explanation for the phenomenon might be due to the larger radiation dose considering the relatively larger volume of the lungs. Meanwhile, the possible reason for out-of-field (low dose area) cancers can be the radiation-induced bystander effect, which is defined as intercellular signaling and tissue inflammation leading to the systemic response that can promote carcinogenesis of organs outside the radiation field (11, 12). This may also be a crucial reason why it is still possible to develop SMNs in low-dose areas. Notably, we did not observe the statistical difference in the distribution of developing pelvic tumors (high dose area) between radiotherapy and non- radiotherapy cohorts. The dose of radiotherapy for pelvic organs is usually higher than that for non-pelvic organs. The possible explanation for the phenomenon was hard to make. The sensitivity of different organs to radiotherapy and the area exposed to radiotherapy might be critical influencing factors leading to this result (13, 14). Total mesorectal excision with adjuvant radiation is considered the standard treatment for locally advanced rectal cancer and obtains a significantly improved prognosis compared with surgery only (15). This may indicate that normal rectal tissue has good tolerance to radiotherapy. In addition, the rectum is located deep in the pelvic cavity, which can also reduce the effect of exposure to radiotherapy to some extent (16).

Another interesting finding is that patients with primary BC show significantly better overall survival than those who develop SMN before and after PSM. Moreover, we separately evaluate the prognosis of patients who develop LAB cancer or hematological malignancies following radiotherapy and obtain similar results. In the SEER cancer registry, subsequent malignant tumors of cancer survivors currently account for 18% of all cancer diagnoses, making it the third most common cancer diagnosis (13). Sanath Kumar had previously proposed the concern about SMN, considering that SMN might lead to a decrease in the OS, although there was still a lack of relevant studies (11). In addition, secondary malignant tumors are bound to require more treatment like chemotherapy and radiotherapy. The treatment of this complex disease is challenging (17, 18). Therefore, it is not surprising that SMNs make a decrease in the OS rate, and this effect is more pronounced in cancer survivors who have a longer survival time.

Statistical considerations are crucial to the interpretation of the results. Some previous studies have used Cox regression analysis to evaluate the risk of SMN (19, 20). However, this method may lead to potential statistical bias because the probability of SMN in BC survivors is relatively low. In this study, the Fine-Gray competing risk regression is utilized to evaluate the risk of SMN, and this method can adequately explore the risk of developing SMN against the competing events, including all-cause death or the last follow-up. In addition, Poisson regression is also performed to make our conclusion more reliable and convincing.

Up to now, this is the first study to evaluate the association between radiotherapy and SMN and reported the effect of SMN on overall survival among BC patients. While radiotherapy brings therapeutic effects, we also need to pay attention to some hidden dangers brought by it (11, 21). More technical improvements may be needed to improve radiotherapy to minimize exposure to normal tissue, especially in the low-dose area. Novel treatment techniques like the scanned beam proton radiation could be considered to reduce the exposure of normal tissues to leaked neutrons (11). In the meantime, image guidance can also be used to direct radiotherapy to reduce the additional accumulation dose of normal tissue (3). Moreover, radiotherapy does not increase the risk of secondary pelvic tumors in patients with bladder cancer, which also provides favorable evidence for the use of radiotherapy in the treatment for BC.

Our study has several limitations that deserve attention when interpreting the results. This study is a retrospective study, and the inherent bias is inevitable. Second, some crucial risk factors related to the occurrence of SMN like smoking, genetic background, living environment, and other cancer-related treatments are lacking in the SEER database (4, 22). These unmeasured covariates may be associated with the development of SMN, and we cannot enroll these related factors into the multivariable risk competing model to balance confounding factors. Then, the SEER database lacks more detailed radiotherapy information such as effects of radiation dose, fractionation, and how RT was used (primary or adjuvant). However, relative homogeneity processing methods for many observation groups have been identified in the SEER database. Meanwhile, the probability of SMN was relatively low, but the true probability of SMN should be higher than the data obtained from our study considering the missed follow-up in the SEER program. Lastly, the SEER database only records the initial treatment, and we lack the follow-up treatment strategy. This may underestimate the risk of radiotherapy for SMN.

## Conclusions

Radiotherapy for BC is associated with SMN. Radiotherapy increases the risk of secondary low-dose area cancer development, including LAB cancer or hematological malignancies. However, this effect is not observed in the high-dose area involving pelvic tumors. In addition, patients developing SMN showed poorer OS than those without. Therefore, more effects should be made to minimize the impact of factors that may increase the risk of SMN after radiotherapy. In the meantime, attention should be paid to the surveillance of SMN in BC patients receiving radiotherapy.

## Data availability statement

The original contributions presented in the study are included in the article/supplementary material. Further inquiries can be directed to the corresponding authors.

## Ethics statement

Ethics approval and written informed consent was not needed for this study as the data is publicly available in the SEER database and it is de-identified.

## Author contributions

RC, XZ and HJ contributed equally to this work and should be considered as co-first authors. XZ was responsible for data collection and analysis. The manuscript was written by the other authors. All authors contributed to the article and approved the submitted version.

## Funding

This work was supported by Grant 2021QNA070 for Youth Research Fund from Fujian Provincial Health Commission, China, and the National Natural Science Foundation of P.R. China (Grant Nos. 81560419, 81960512, and 81760457).

## Conflict of interest

The authors declare that the research was conducted in the absence of any commercial or financial relationships that could be construed as a potential conflict of interest.

## Publisher’s note

All claims expressed in this article are solely those of the authors and do not necessarily represent those of their affiliated organizations, or those of the publisher, the editors and the reviewers. Any product that may be evaluated in this article, or claim that may be made by its manufacturer, is not guaranteed or endorsed by the publisher.
